# Children’s views on weight measurement and talking about weight

**DOI:** 10.1186/s12889-025-22354-7

**Published:** 2025-04-04

**Authors:** Ryan Herbert, Fiona Gillison, Elisabeth Grey, Abigail Hewitt, Alice Woods, Charlotte Jackson, Grace Wisbey, Jack Perks, Maddy Longhurst

**Affiliations:** 1 Centre for Motivation and Health Behaviour Change, University of Bath, Bath, BA2 7AY UK; 2 The National Institute for Health and Care Research Applied Research Collaboration West (NIHR ARC West), University Hospitals Bristol and Weston NHS Foundation Trust, 9 Floor, BristolWhitefriars, Lewins Mead BS1 2NTBristol UK; 3 Bristol Medical School, Population Health Sciences, University of Bristol, Bristol, UK

**Keywords:** Children, Weight communication, Weight management, Views, Family-centred approach, Health promotion

## Abstract

**Background:**

Parents are considered important in promoting children’s healthy weight, but express concern about how to talk to children about weight without harming their wellbeing. However, there is little research with children themselves as to what they expect and want in relation to managing their weight. This study aimed to explore children’s views about weight measurement, and whether and how information about their weight should be shared with them.

**Methods:**

Primary school-aged children were recruited through their school to take part in focus groups. Discussions were focused around two tasks: drawing a shared mind map of sources of information about weight; and exploring responses to a structured story about a child’s experience of being weighed and measured. The discussions were recorded, transcribed and analysed using thematic analysis.

**Results:**

Forty-eight children took part, across eight focus groups. Theme 1 explored how children considered that most weight discussions were peripheral to them, often overheard conversations related to family members’ attempts at weight loss. Theme 2 showed how children empathised with the challenges parents face when deciding whether to disclose weight status, in balancing the desire to avoid upsetting children with the desire to be honest and action focussed. Theme 3 summarised children’s expectations about what should happen if children are found to have overweight or obesity, emphasising a whole-family approach.

**Conclusion:**

Children’s views about how and whether to talk about weight varied, but appeared underpinned by the awareness of stigma and belief in individual responsibility and capability for controlling body weight.

## Background

Childhood obesity remains a public health challenge, associated with excess weight in adulthood increasing the risk of non-communicable disease risk and premature mortality [1]. Children living with obesity also experience poor mental health and well-being [2]. The determinants of childhood obesity are multi-faceted, with rising prevalence reflecting significant changes to the food, social and physical environment [3]. While there is increased recognition of the need to consider the multiple levels of influence in determining obesity (e.g., whole systems approach) [4], most interventions still focus on individual behaviour change, whether of children, parents or both [5].

Parents are important in promoting a healthy childhood weight, so assisting them to better support their children in ways that parents find acceptable is vital [6]. In some countries screening programmes take place, such as the National Child Measurement Programme in England (NCMP) [7], in which parents are provided with feedback about their child’s weight [7–9]. Parents often underestimate their children's weight status, making unsolicited feedback from such programmes surprising and sometimes unwelcome [10, 11]. Further, providing feedback in this way with limited engagement with healthcare professionals to facilitate discussion [7, 8] has yet to be shown to be effective [9]. Many parents raise concerns about the negative impact that weight measurement could have on their child’s self-esteem and well-being [12, 13] and highlight fear that making children aware of their weight status, and talking to them about weight, may expose them to weight stigma. While research investigating the impact of parent–child weight communication indicates that it is not inevitable that talking to children about weight has a negative impact [14], negative forms of communication, like criticising or teasing children about their weight and encouraging children to lose weight (as opposed to encouraging healthy behaviours in general) can have adverse effects on their well-being [14, 15].

Children's perspectives are frequently omitted in research on weighing and measuring processes, meaning much of the guidance and understanding around the process does not incorporate their views [16, 17]. Most research reports on parents’ views [11, 12], and both parents and healthcare professionals report an interest in learning more about what children think [17]. One study has been published reporting on 21 children’s views following weight measurement in 2012, showing mixed views of the experience, and concerns from children resulting from whether and how parents had provided feedback [18], but little else is available. Therefore, the aim of this study was to explore children’s perceptions about the need to measure their weight, and whether and how this information should be shared with them.

## Methods

Ethical approval was obtained from the University's Research Ethics Approval Committee for Health (REACH) (Ref: EP 22 102).

### Study design

A qualitative phenomenological design was adopted, using focus groups to facilitate in-depth discussions, allowing children to listen and respond to each other’s attitudes, concerns, and opinions [19].

### Participants

Eligible participants were children aged 8–11 (school years 4–6); this age range was chosen to reflect the views of children at or around the age at which they would typically take part in the National Child Measurement Programme (NCMP) in England (i.e., Year 6) [7]. Children could participate regardless of their weight status.

A list of all primary and secondary schools in four local authorities in the Southwest of England was obtained through the GOV.UK [20] school finder tool. Children in specialist schools were excluded as the majority of these schools do not participate in the NCMP [7]. Two schools outside the Southwest of England (Hampshire and East London) were invited from the research team’s networks after local options had been exhausted. We aimed to conduct 8 focus groups with 4–6 students in each to provide a breadth of views, mindful of both (a) the difficulty of recruiting children in a stigmatised research area, and (b) the need to ensure these were of sufficient quality as much as ensuring we reached a certain quantity [21].

### Procedure

Invitations were emailed to head teachers providing information about the study and a copy of guidance for parents on talking about weight (to show the type of resource the research could inform) [22]. Reminders were sent 1–2 weeks later. Interested schools were asked to distribute letters or emails to parents of eligible students, enclosing an information sheet, links to a video version of the information suitable for children, and a consent form. Written, opt-in consent from a parent or carer was required for any child to take part.; details of the child’s ethnicity and gender were requested on the consent form. Weight and height measurements were not required. Schools collated completed consent forms on behalf of the research team according to their usual procedures for seeking parent permission and provided these to the Researchers on the day of the focus groups. The Researchers checked a consent form had been received for each student attending prior to starting the focus group.

Focus groups took place in schools during the school day, co-led by two research assistants (RAs), supported by the lead facilitator (FG). The lead facilitator is a qualified and experienced health psychologist, and the RAs were undergraduate researchers trained in qualitative methods (n = 7; 2 males and 5 females rotating between focus groups) and in preparation for the focus groups the RAs undertook an e-learning module on the risks and best practices to adopt for talking to children about weight [23]. Each school’s safeguarding policy was adhered to, and consequently in three of four schools a teacher or teaching assistant was present during the session but took no active part.

### Focus group design

At the start of the focus group, children were briefed on the study's purpose and asked for assent, with the option to withdraw at any time. They were asked not to talk about their own weight or that of anyone else in the focus group during the session and were provided with details of sources for further support to take away.

To maximise children's enjoyment and minimise potential weight concerns [24] discussions focused around two main activities: 1. Creating a collaborative mind map about where children hear about body weight, discussing their trust in each source; 2. Responding to a story about a child's experience with the NCMP [17]. This included consideration of whether children should be informed of their weight, and if so how. At the end of the focus group, children independently wrote down their final thoughts and placed them in a sealed box (secret box approach) [25]. This allowed additional input, especially from those who may not have felt confident or had the opportunity to share their thoughts in the group setting. Focus groups were recorded, transcribed verbatim and anonymised for analysis.

### Analysis

Focus group transcripts were analysed using thematic analysis [26]. A pragmatic approach was taken adopting Braun and Clarke’s six-step framework: data familiarisation, initial coding, searching for themes, reviewing themes, defining, and naming themes and producing the report. The analysis was undertaken by the principal investigator (FG), who undertook the inductive coding of all transcripts and development of themes. The RAs who assisted with data collection were undergraduate students who had received training in qualitative research methods as part of their course. They also familiarised themselves with all transcripts and contributed to discussion around the interpretation of the focus groups and potential themes prior to the coding process. Written data (i.e., mind maps and individual written submissions) were collated and the frequency in which concepts occurred was used to support the thematic analysis.

## Results

Of 285 primary schools approached, 11 agreed to take part. Written parental consent forms were sufficient to run focus groups in four of these. Eight separate focus groups were run, involving 48 children primarily in Years 5 and 6 (ages 9–11; Table 1). Most participants (65%) identified as White British, 2% as Black British, 2% as mixed ethnicity; 31% did not disclose their ethnic identity. Focus groups ran for an average of 27.2 min (SD = 3.5 min).

**Table 1:** Demographic detail of focus groups

	School 1		School 2	School 3		School 4		
Ethnicity (n)	White British (5) Black British (1) Not stated (2)		White British (6)	White British (12) Not stated (7)		White British (8) Mixed ethnicity (1) Not stated (6)		
	FG1	FG2	FG3	FG4	FG5	FG6	FG7	FG8
Gender (n)								
Male	2	2	5	5	5	2	3	1
Female	2	2	1	5	4	4	2	3
Participant School Year (n)	Y6	Y4 (2) Y5 (2)	Y5 (1) Y6 (4) Y7 (1)	Y6	Y6	Y5	Y5	Y5
IMD decile^a^	3	3	10^b^	5	5	10	10	10

Key: FG—Focus group, IMD—Index of multiple deprivation [27]. Y4 includes children aged 8–9 years old, Y5 9–10 years, and Y6 10–11 years. ^a^Decile 3 indicates the 20–30% most deprived areas, Decile 5 the 40–50% most deprived, and Decile 10 the 10% least deprived areas. ^b^All schools were state run primary schools except for FG3 which was fee paying

A summary of the sources of information about body weight that children reported across focus groups and additional information provided in individual submissions that were not volunteered during focus group discussions is presented in Table 2. Three themes were developed to summarise children’s inputs: (1) visibility of weight talk; (2) the impact of being told you are overweight; and (3) acceptability of weight surveillance and management.

**Table 2:** Sources of information about weight reported in Activity 1

Source	Home	School	Online	Health service	Family
	Books (2) TV (including adverts, Joe Wicks, News) (4) Club (sports, scouts)^b^	PHSE (4) Science (2) Life Skills (1) PE (1) Friends (1) Assembly (1) Maths (1)	Google/internet (4) Adverts online (1) YouTube (4) Gaming (1) Snapchat (1) TikTok (1)	Doctors^a^(2) Posters in doctor/ dentist (1)	Parents (direct) (3) Overheard conversations (3) Mealtime discussions (1) Wider family members (siblings, grandparents, cousins) (2)

^a^participants mentioned doctors as people they could ask, but not as people they had directly heard from; ^b^children did not all receive weight information from the same sources, in this example, some contested whether this should be included as many clubs were not sources of information about weight

### Theme 1: Visibility of weight talk

At the start of the focus groups, most children struggled to add ideas to the mind map, as they did not remember directly encountering any discussions about body weight. However, after prompting about a range of potential sources (e.g., *Do you hear about it at school, at home?*), some children reported that they felt body weight was an issue peripheral to them and largely a discussion between adults (“*It’s just adults talking to adults, and I just happen to overhear.*”, Boy 4, FG5). In two focus groups, children stated they find adults talking about weight very ‘boring’ and typically do not listen.

*Whenever they try to speak about it [weight] I just go upstairs to my room and shut the door cause they will speak about it for like, 2 hours. *(Boy 3, FG7)

Where children did report hearing about body weight, this was commonly about family members trying to lose weight:

*When I go to [stay with] my family […] lots of them are overweight and they say like “Oh my gosh I wanna lose weight so bad like I don’t feel like right about my body” and stuff like that.* (Girl 1, FG2)

*Oh yeah, erm, [I hear about] intermittent fasting […] my mum and dad did erm, not an intermittent fast, just did a fast for about a week..* (Boy 1, FG4)

Only in very few cases did children report parents or others actively engaging with them in discussions about health behaviours or healthy weight, for example;

*Sometimes they [school] just erm they talk about weight and some things that er we need to know for when we grow up.* (Girl 2, FG1)

*Urm so my dad likes taking me on bike rides and he says like it’s good for your system and stuff like that, and my dad also has a blender and he says it’s good for your nutrients and he puts lots of fruits in it.* (Girl 1, FG2)

Children also picked up social cues about weight through television and social media. This was rarely information they sought themselves but reflected what they were exposed to through activities with families or gaming. Several children also mentioned adverts encouraging people to buy things to help them lose weight.

*Um sometimes I watch it on Youtube or something and there are adverts like “lose weight by eating duh-duh-duh”.* (Boy 1, FG6)

*I think it was Piers Morgan [TV broadcaster] who said that telling people they are overweight can be a good thing so they can have a bit of motivation.* (Boy 2, FG3)

Overall, children exhibited a cautious approach when engaging with information on social media and demonstrated an awareness of the potential for misinformation and digital manipulation by users. Consequently, the majority of children expressed scepticism towards the content they encountered, recognising their difficulty in discerning between authentic and fabricated information.

*The internet isn’t very trustworthy because you get lots of lies and choice, so you don't know what to believe or not?* (Girl 3, FG8)

*Especially on like videos on TikTok and stuff, people like put on filters* (Girl 1, FG2)

Participants also reported a few examples of weight within fictional books or films, where being overweight seemed to always be presented in a very negative, or cautionary way. For instance, we visited School 4 shortly after National Book Day, children volunteered the example of Harry Potter's cousin Dudley as a source of weight information; noting he is very overweight, which is associated in the story with negative personality traits like greed and spitefulness.

### Theme 2: The impact of being told you’re overweight

Children in all focus groups recognised the challenges parents face in deciding whether or not to tell children if they are overweight. Most children’s initial response was that they should be told, but as the story unfolded during the second focus group activity, they began to debate the pros and cons, and very few children held firm or consistent opinions. The perspectives they introduced in support of parents telling children included the importance of being honest with children (“*Cos if you found out and looked at the letter and found out, that’s a bit untrustworthy like why did you lie to me*?”, Boy 4, FG5), children’s right to know information about themselves (“*because it’s her weight, she can do what she wants with it.”*, Girl 2, FG6) and believing that children would want and be able to take action if aware (“*Sam should tell her because if urm if Sam tells urm the girl she will lose weight and she will eat healthier like salad and …..*, Boy 2, FG2).

In contrast, participants felt parents may choose not to tell their children about their weight status as they may be “*hurt*”, “*angry*”, “*sad*”, “*upset*”, “*depressed*” or “*disappointed*” that they are overweight *(Cos it might like upset her and give her insecurities;* Boy 4, FG5), and could see that there may be consequences of these feelings;

*I think that she [the girl in the story] would have like, she would have been upset but then adding on from that she would have tried to like look better and stuff like that and she might not have gone to school cos she would have been really sad. (Girl 1, FG2)*

One child also expressed how hearing from a parent would be different from teasing about weight by peers*;*

*Let’s say Aisha’s getting picked on, and she tries not to believe that she’s really overweight, but people are just picking on her because they are mean. But if her father told her “you’re overweight” then she would really believe it. *(Boy 2, FG3)

Parents were trusted both as a source of information and to know what changes to make (*I would trust my parents but not fully my friends cause friends like, kids, kids don’t really know all the correct information*, Boy 1, FG2). In imagining themselves in the role of a parent, children came up with some nuanced ideas of how parents could acceptably approach this:

*I don’t know, I don’t think I’d tell her actually. I think I might cook her healthy dinners and take it into my own hands.* (Boy 2, FG3)

Nonetheless, even when the group had reached a point of agreement that it was best not to tell children, just to make changes as a family, others could spot reasons why this may not work with children of their age:

*I would think like I could properly catch on to like them doing this. It’s like obvious, putting on a run every single weekend I would […] Yeah, start to catch onto it.* (Boy 2, FG1)

Children expressed clear ideas about how parents should tell their children if they chose to do so, including telling children slowly, using sensitive language, and being calm and kind.

### Theme 3: Acceptability of weight surveillance and management

There was widespread acceptability of weight measurement among participants, who universally believed that measurements are taken for health reasons (“*because if you have lots of weight you will have like trouble breathing and stuff*.” Child 3, FG5). In past work, parents have expressed concern that the concern is partly aesthetic (to make children “look a certain way”) [28], but this was not discussed explicitly in any focus group, beyond a comment from one child suggesting children may feel the need to “*look better*” if informed they are overweight (Girl 1, FG2).

It was consistently seen across focus groups that the appropriate response when a child is found to be overweight, is to help them in, and hold them partially responsible for, reaching a healthy weight. This reflected an individualistic view that the development of being overweight results from eating too much, or less healthy foods (junk food), and not doing enough exercise, and the associated belief that it is within the power of parents and children to change this:

*Don't just sit around doing nothing. Get out do some exercise, but it's also okay to like watch some telly and like play on the Switch […] You're allowed to do it, but just don't do it like all the time. Yeah, do it occasionally.* (Boy 1, FG8)

This belief did not seem affected by children’s observations that adults’ weight loss attempts were often unsuccessful. Thus, children in all focus groups agreed that regardless of whether or not a child was told about their weight, parents should take action as a whole family to be healthier (eat better, take more exercise):

*Maybe they could go on lots of walks, like the whole family. So they can like, so if she doesn't feel like it's just her *(Girl 2, FG6)

## Discussion

This study provides insight into children’s views about why they are weighed and what should happen if they are found to be overweight, with a particular emphasis on their expectations of parents. In Theme 1, children noted their limited exposure to and engagement with discussions about body weight with adults. They primarily learned about weight through overhearing adult conversations, observing others’ weight-loss efforts, and inferring messages from advertisements (usually about weight loss). Theme 2 reported participants’ nuanced understanding of their parents' perspective, acknowledging the potential for upsetting children and how this could be minimised. Interestingly, participants seemed largely tolerant of some degree of deception by parents if this was to avoid upset. Theme 3 reflected children’s understanding of weight as a personal responsibility and their expectation that being categorised as overweight would lead to behaviour changes to reduce excess weight for their health, ideally as a family. Overall, children were aware of weight stigma and implicit messages around weight but did not reach a consensus on whether parents should explicitly discuss weight with their children.

Few children reported having had direct conversations about weight or its determinants with their parents, but the majority were familiar with observing adults engaging in weight management behaviours, whether within their family or in the media. These findings align with prior research [29], highlighting the impact of role models, particularly parents, on children's understanding of weight-related actions. However, given the risks of some forms of incidental parent–child communication about weight [28], better guidance for parents on how to avoid negative outcomes may be needed to ensure this is framed and delivered positively [14]. Some guidance has been developed [22] and found to be acceptable to parents and school nurses [30] but is yet to be widely implemented in practice. In its absence, the lack of direct engagement seemed to result in children adopting implicit messages from their families' attitudes and behaviours around weight.

Children talked about other direct and indirect societal influences, apart from their parents, such as books and media that contribute to their understanding of weight. As children grow older, they internalise these messages and incorporate them into their own understanding [31]. Recent research highlights a prevailing narrative in children's stories, be it in books or on screen, where body weight serves as a shorthand for negative traits, associating characters' weight with unfavourable attributes [32]. Exposure to social or online media was limited in this age group, yet children still discussed advertisements and websites as sources of weight information. Most children knew these were not trustworthy sources, but past work suggests that children increasingly seek and trust scientific information from the internet [33].

Future work would be useful in finding ways to share information with parents about the potential consequences of *not* engaging with children in conversations about weight (i.e., that the vacuum will be filled by their own observations and access to media), that do not attribute blame to parents or negatively impact their wellbeing. At a societal level, this could involve shifting public understanding of the determinants of weight and health from a primarily individualistic view, to accepting the powerful wider environmental and societal determinants of weight [34, 35] and health [36]. These includes the commercial determinants of health, such as unhealthy commodities and marketing and business practices, which are an important contributor to the current obesogenic environment but often poorly understood by the public [37]. Sustainably changing public understanding of these wider determinants of health has proven challenging [38], so warrants further research. In the meantime, when sharing insights with parents at an individual level, this message may be better communicated through personal interactions with health care professionals, rather than through letters or mass media. A one-to-one setting provides an opportunity for nuance and tailored discussion on the options open to parents, which may promote more constructive outcomes for families and reduce the risk of commonly reported negative responses to national mail-based programmes [11, 12].

Theme 2 highlights how children of primary school age understand that there is stigma to being overweight. Participants recognised that being told they are overweight could trigger negative emotions, mirroring parental concerns about upsetting children and affecting their self-concept [12, 13], leading to some acceptance of parents taking action without letting their child know. However, there is a disparity between children's belief in their parents' ability to help them achieve a healthy weight, as reported elsewhere [39], and parents' reported low self-efficacy and outcome expectancies [40]. As such, parents’ attempts to try and address children’s weight may need to incorporate how they will manage children’s expectations of achieving healthy weight.

If children retain the belief that weight loss is simple and merely a decision, it could exacerbate the perceived stigma of staying overweight during childhood [41, 42]. Future research could explore whether there are ways to counteract this messaging for children, for example as part of the educational curriculum in schools. In most focus groups conducted as part of our study, children recognised school as a trustworthy source of weight information but rarely remembered learning about weight at school. Despite schools having a responsibility for promoting children's health and wellbeing in the United Kingdom [43], teachers are often uncomfortable or unconfident in discussing body weight [44]. As part of a systems-wide approach to reducing weight stigma, future research could explore the feasibility and appropriateness of schools taking a more active role in shifting children’s understanding of body weight determinants, as part of their efforts to reduce weight-related teasing and bullying [45]. This may include investigating the extent to which schools use, or rely on commercially sponsored of educational materials around health that endorse an individualistic approach, and seeking to provide alternatives [46, 47].

### Limitations

As anticipated given the sensitive topic, our recruitment rate was low, likely attracting families already interested in health and less concerned about weight conversations (i.e., a likely self-selection bias). This study represents the views of many children but lacks geographic and ethnic diversity. We purposefully did not record participants' weight status, so we cannot assess the representation of children living with overweight and obesity. The focus groups were effective in generating discussion, but managing behaviour in the largest groups was challenging, with overlapping conversations possibly hindering some children from contributing fully and reducing time for focused discussions. At the school’s discretion, focus groups were mixed gender, and it may be that some children were hesitant to share their views among peers, and particularly among peers of a different gender.

## Conclusion

Primary school-aged children in this study had a similar understanding of what it means to be overweight, and the pros and cons of talking to children about their weight, as has been reported by parents. They believed in avoiding unnecessary weight communication with children to avoid upset, and favoured a whole-family approach if action was to be taken. Participants reported little direct experience with weight communication intended for their level of understanding, instead getting their information from sources such as overheard adult conversations, adverts, books, and TV. They exhibited an individualistic understanding of the determinants of weight and an optimistic belief of how easy it is for children to reach a healthy weight.

## Data Availability

The datasets generated during and/or analysed during the current study are available from the corresponding author on reasonable request.

## Abbreviations

NCMP: National Child Management Program
RA: Research assistants
